# Report of the Buffalo General Hospital

**Published:** 1865-08

**Authors:** J. R. Lothrop


ART. II.—*Report of the Buffalo General Hospital.* By J. R. Lothrop, M. D.

This Report embraces a period of one year, viz., from July 1st, 1864, to July 1st, 1865, and includes all patients treated by the several attending physicians and surgeons.

| | |
|---|---|
| Number of Patients received during the year | 894 |
| " not placed under treatment | 6 |
| | 888 |

The results of treatment may be stated as follows:

| | |
|---|---|
| Discharged well | 594 |
| " relieved | 148 |
| " not relieved | 30 |
| Died | 33 |
| Remaining under treatment | 83 |
| | 888 |

From this it appears that the ratio of deaths has been a little more than 3 per cent., or about 1 in 27.

The cases received and placed under treatment admit of the following general classification:

SURGICAL CASES.

| | | | |
|---|---|---|---|
| Abscess | 3 | Lupus | 1 |
| Amputations | 28 | Necrosis | 2 |
| Affections of the Eye | 17 | Orchitis | 6 |
| Concussion of the Brain | 1 | Paralysis | 11 |
| Cancer | 4 | Paronychia | 1 |
| Caries | 3 | Prolapsus Uteri | 1 |
| Erysipelas | 4 | Retention of Urine | 3 |
| Fractures | 15 | Synovitis of Knee | 1 |
| Fistula in Ano | 1 | Tonsillitis | 4 |
| " Vesico Vaginal | 1 | Tumors | 4 |
| Frost Bite | 6 | Ulcers | 12 |
| Hæmorrhoids | 4 | Stricture Urethral | 3 |
| Hernia Inguinal | 1 | Varicocele | 1 |
| Hospital Gangrene | 33 | Varicose Veins | 4 |
| Inflammation of Ear | 2 | Venereal Diseases | 15 |
| Injuries, (various,) | 52 | Gun-Shot Wounds | 221 |

MEDICAL CASES.

| | | | |
|---|---|---|---|
| Albuminuria | 1 | Diseases of Heart | 8 |
| Alcoholismus | 1 | Insanity | 1 |
| Asthma | 3 | Jaundice | 5 |
| Bronchitis | 15 | Neuralgia | 2 |
| Chorea | 1 | Nephritis | 1 |
| Cholera Morbus | 1 | Old Age | 1 |
| Congestion of Brain | 1 | Parturition | 3 |
| Coup de Soleil | 4 | Peritonitis | 1 |
| Debility | 52 | Phthisis | 35 |
| Delirium Tremens | 6 | Pleuritis | 6 |
| Diarrhœa, Chronic | 141 | Pneumonia | 7 |
| Dropsy | 3 | Rheumatism, Acute | 3 |
| Dysentery | 11 | Rheumatism, Chronic | 40 |
| Epilepsy | 4 | Rubeola | 1 |
| Fever, Intermittent | 30 | Scarlatina | 1 |
| " Remittent | 11 | Sciatica | 2 |
| " Typhoid | 6 | Variola | 3 |

This Hospital having been one designated by the War Depart, ment for the reception of sick and wounded soldiers, will readily explain the large number of cases of gun-shot wounds and chronic diarrhœa. A purely civil hospital would not be likely to receive so great a number of such cases. These groupings, as given above, are in some cases too general, and need more detailed statement. It will be seen, debility covers a large number, in which a definite diagnosis was not or could not be arrived at, making the statistics somewhat indefinite and less valuable. All such grouping is objectionable, and should be, as much as possible, avoided. Of course, in many cases an autopsy is the only thing which will clear up satisfactorily an obscure case, but modern methods of diagnosis are competent to make, if well employed, a more definite classification. The same objection lies against so general a term as dropsy, though it is very probable that it is used as but another term for ascites.

CAUSES OF DEATH.

| | | | |
|---|---|---|---|
| Abscess, Psoas | 1 | Fracture, Foot and Ankle, compound (after Amputation) | 1 |
| Cancer of Uterus | 1 | Hospital Gangrene | 3 |
| Concussion of Brain | 1 | Old Age | 1 |
| Debility | 2 | Pericarditis | 1 |
| Delirium Tremens | 1 | Peritonitis | 1 |
| Diarrœha, Chronic | 4 | Phthisis | 6 |
| Dropsy | 1 | Pleuritis, (1 empyæma,) | 2 |
| Dysentery | 2 | Pneumonia | 2 |
| Epilepsy, (after trephining,) | 1 | Pyæmia | 2 |
| Fever, Typhoid | 2 | Valvular disease of Heart | 1 |
| Fracture of Skull | 1 | | |

*Amputations.*—Twenty-eight amputations are recorded—without explanation this would convey a wrong impression. The amputations were not all made at the Hospital. In most cases, this had been done before the patients were admitted. The cases were mostly soldiers, some of whom had undergone the operation on the field, and some in other Hospitals. They were, therefore, received for treatment after amputation. The cases of amputation actually performed at the Hospital, will be spoken of separately. With this explanation, the cases of amputation were—of the thigh, four; of the arm and forearm, nine; of the leg, ten; of the foot, one; of fingers, four.

*Cancer.*—The cases of cancer were, of the breast schirrus, one, removed; one of the os uteri causing death, in a woman about 45 years of age; one of the testis, medullary, in a young man, thirty years, removed; and lastly, a medullary cancer on the outer and upper part of the thigh in a man about 50, which was not interfered with, as it had so far involved the neighboring tissues as to offer no prospect of benefit by removal.

*Diseases of the Eye.*—Conjunctivitis, nine; ectropion, two; melanosis, one; injury (gun-shot) causing loss of sight in both eyes, one; iritis one, catarrhal ophthalmia, one; opacity of the cornea, two.

*Fractures.*—The fractures were of the clavicle middle third, one; lower jaw comminuted, one; femur middle third, one; fibula alone, one; tibia alone, one; tibia and fibula both, three; humerus, two; patella, two; ribs, two; skull, one. The fracture of the clavicle was caused by a direct blow upon the shoulder, and there being considerable obliquity and overlapping, the result was rather more than the average deformity, though the pad and sling were employed, and the patient made very uncomfortable by the complicated retentive apparatus. The fracture of the femur was treated by extension by means of weights and without a long side splint; the leg and thigh being propped by pads. Short splints were employed. The counter extension was obtained by elevating the lower end of the bed by means of a block three or four inches in thickness. In addition, a board foundation upon which the bed rested was hinged near the middle, so that both feet and head could be elevated. On the inclined plane thus formed, at the lower end, the weight of the body gave sufficient counter extension. The weight employed never exceeded thirteen pounds, and was for the greater part of the time less than ten pounds. It was attached to a cord which played over a pulley fastened by means of a frame to the foot of the bed. The result was satisfactory, the shortening being less than half an inch. This apparatus proved more comfortable to the patient and more easily managed, than any heretofore employed.

*Paralysis.*—Of these, six were cases of paraplegia, four of hemiplegia, one of paralysis of the facial nerve which was caused by exposure to cold air.

These cases of paraplegia illustrated some of the points of differential diagnosis. One was in connection with angular curvature or Pott's disease, and in this case the control of the sphincters was not lost, confirming the statement of Romberg that paralysis from affection of the bones of the vertebral column spares the sphincters. In another in which the cause was myelitis, the control of the sphincters was lost, and there was an entire loss of sensation, motion, and of the reflex function, as well as great diminution of temperature; but twitchings and spasmodic movements were frequent. In another, a case of meningitis with effusion, the characteristic rigidity of the muscles of the back, increased at times by spasmodic action was exhibited in most marked degree.

*Venereal Diseases.*—Under this head are included four cases of primary, nine cases of secondary syphilis, and two cases of gonorrhœa.

*Gun-shot Wounds.*—The two hundred and twenty-one cases of gun-shot wounds were of such a nature as to admit of removal, therefore generally light, or so far progressed as to offer no obstacle to a journey from camp hospitals or regular military hospitals near the front. Some were received soon after the infliction of the wound, while others had been some time in hospitals elsewhere, and were sent away to make room for more recent and severe cases. The severe battles in the summer of '64 around Richmond, furnished a good many cases of recent wounds—the wounded being sent directly from the field to the hospital. Such cases were generally light wounds of the extremities, but in many cases from delay and heat, and the motion unavoidable in transportation a long distance, the wounds became inflamed, and if not actually gangrenous, were attacked by gangrene soon after arrival. The results were, therefore, very serious in many cases of originally light wounds. Of the two hundred and twenty-one cases of wounds, seventy-two were of the upper extremities, and one hundred and fifteen of the lower extremities. Almost all cases of wounds of the head and trunk were from other hospitals, and were not of recent date. They were therefore convalescent. Among these wounded, thirty-two cases of hospital gangrene occurred, and three terminated fatally. Those who recovered were in many cases more crippled than was due to the nature of the primary wound.

In the local treatment of gangrene, turpentine, bromine, and permanganate of potash were the remedies most employed. The latter remedy appeared more positively beneficial than the others, and preference was given to it. But in many cases, as the experience of others has taught, for a time no remedy seemed to exert any decided influence. The measure apparently most useful was removal from the hospital building to tents in the yard. The benefits of this measure were soon apparent. The two important objects requisite, fresh air and isolation, were thereby secured.

The medical cases cannot be given in greater detail from lack of knowledge. In some cases the statement of diseases is too general, and therefore likely to create an unjust impression of the nicety of the diagnosis. For instance, eight cases of disease of the heart are given, in a general way, when upon examination it is found that this embraces five cases of valvular disease, two of hypertrophy, and one of pericarditis. The means are not at hand to make such a statement, as will set forth clearly, the differential diagnosis, which was probably made in cases embraced in a class.

It cannot escape observation that the success in the treatment of chronic diarrhœa was quite remarkable. Of the one hundred and forty-one cases, fifteen were under treatment at the close of the year, and the results therefore not determined. Excluding these one hundred twenty-six remain, in which results are reported. Of these one hundred and twenty-six, one hundred and eight recovered, and were discharged well; sixteen were much relieved, and two only ended in death. One familiar with cases of chronic diarrhœa, contracted in the camp, or in the field, is fully aware of its often intractable nature, and the slowness of its progress, i. e. towards recovery. In treating such cases, we must be prepared to meet with ill success, for often remedies exert no beneficial influence, and in spite of all measures the disease steadily goes on to a fatal termination. Autopsies explain it by revealing a thickened, contracted and softened colon, and the lower portion of the small intestine inflamed, reddened, and having undergone a change or destruction of tissue. The treatment adopted in these cases was more largely dietetic and hygienic than medicinal. The astringent preparations of iron were more employed than any other medicines, and particularly the muriated tincture of iron. Continued for a long time more benefit was perceptible from it than from any other medicine. When the absorbing power of the intestine is not so much interfered with that nutrition is defective, recovery will usually follow when sufficient nourishing food can be obtained. The results in these cases will compare favorably with those of treatment in any other hospital.

